# Genotype-Phenotype Characterization of Novel Variants in Six Italian Patients with Familial Exudative Vitreoretinopathy

**DOI:** 10.1155/2017/3080245

**Published:** 2017-07-05

**Authors:** Giancarlo Iarossi, Matteo Bertelli, Paolo Enrico Maltese, Elena Gusson, Giorgio Marchini, Alice Bruson, Sabrina Benedetti, Sabrina Volpetti, Gino Catena, Luca Buzzonetti, Lucia Ziccardi

**Affiliations:** ^1^Department of Ophthalmology, Bambino Gesù IRCCS Children's Hospital, Rome, Italy; ^2^MAGI-Human Medical Genetics Institute, Bolzano, Italy; ^3^MAGI-Human Medical Genetics Institute, Rovereto, Italy; ^4^Eye Clinic, Department of Neurosciences, Biomedicine and Movement, University and AOUI (Azienda Ospedaliera Universitaria Integrata) of Verona, Verona, Italy; ^5^Dipartimento Anestesia e Rianimazione Materno Infantile, Ospedale San Filippo Neri, Rome, Italy; ^6^“G.B. Bietti” Foundation, IRCCS, Rome, Italy

## Abstract

Familial exudative vitreoretinopathy (FEVR) is a complex disorder characterized by incomplete development of the retinal vasculature. Here, we report the results obtained on the spectrum of genetic variations and correlated phenotypes found in a cohort of Italian FEVR patients. Eight probands (age range 7–19 years) were assessed by genetic analysis and comprehensive age-appropriate ophthalmic examination. Genetic testing investigated the genes most widely associated in literature with FEVR: *FZD4*, *LRP5*, *TSPAN12*, and *NDP*. Clinical and genetic evaluations were extended to relatives of probands positive to genetic testing. Six out of eight probands (75%) showed a genetic variation probably related to the phenotype. We identified four novel genetic variants, one variant already described in association with Norrie disease and one previously described linked to autosomal dominant FEVR. Pedigree analysis of patients led to the classification of four autosomal dominant cases of FEVR (caused by *FZD4* and *TSPAN12* variants) and two X-linked FEVR probands (*NDP* variants). None of the patients showed variants in the *LRP5* gene. This study represents the largest cohort study in Italian FEVR patients. Our findings are in agreement with the previous literature confirming that FEVR is a clinically and genetically heterogeneous retinal disorder, even when it manifests in the same family.

## 1. Introduction

Familial exudative vitreoretinopathy (FEVR, OMIM 133780) is a complex disorder characterized by incomplete development of the retinal vasculature. Typical signs of this disorder include avascular areas in the peripheral retina detectable by fluorescein angiography due to abnormal congenital angiogenesis that can cause early-onset neovascularization, falciform folds, lipid exudation, and tractional retinal detachment [1, 2].

Insights into genetic causes of this disorder have emerged in the last few years. Four genes involved in the Norrin/*β*-catenin signaling pathway are linked to the disease: *NDP*, *FDZ4*, *LRP5*, and *TSPAN12*, and are responsible for about 50% of FEVR cases [3].

Norrin/Frizzled signaling is known to be critical for retinal angiogenesis by controlling retinal vascular growth and architecture both in the developing eye and in adult vasculature; impairment of the signaling system has profound effects on retinal vascular organization and leads to severe vascular abnormalities [4, 5].

Depending on which gene is defective, FEVR may have different patterns of inheritance. Autosomal dominant inheritance, due to *FZD4*, *LRP5*, and *TSPAN12* gene variations [6–8], is prevalent. Autosomal recessive and X-linked inheritances have also been reported due to variations in the *LRP5* and *NDP* genes, respectively [9, 10]. Recessive variants in *TSPAN12* have also been described in a family with retinal dysplasia and severe FEVR [11].

Clinical features are extremely variable, ranging from absence of symptoms to complete blindness, even in the same family [12].

This evidence has led researchers to suspect the involvement of other unknown genes [3, 8]. Two other genes, *ZNF408* encoding zinc finger protein 408 and *KIF11* encoding kinesin family member 11, are linked to FEVR, although the evidence of their association needs to be clarified. When genetic testing was performed on the patients enclosed in the present study, the above-mentioned novel genes had not yet been discovered; therefore, we only focused on testing for the *NDP*, *FDZ4*, *LRP5*, and *TSPAN12* genes.

In literature on Italian FEVR patients, only three genetic association studies describing a total of six probands are reported [13–15]. Here, we present the results of clinical and genetic characterization of the largest Italian FEVR population, consisting of six probands and ten relatives, as a contribution to the FEVR disease molecular epidemiology in our country.

We identified four new variants, one variant already described in association with Norrie disease and a variant previously described in association with autosomal dominant FEVR. The genotype-phenotype correlation of each variant was evaluated by a family segregation study, and new variants were further characterized for their putative pathogenic potential by in silico evaluation.

## 2. Materials and Methods

Probands (age ranging from 7 to 19 years) and their families were examined in two different eye clinics: the Eye Clinic, Department of Neurosciences, Biomedicine and Movement, (University and Azienda Ospedaliera Universitaria Integrata of Verona, Italy) and the Department of Ophthalmology (Bambino Gesù IRCCS Children's Hospital, Rome, Italy).

Probands and relatives underwent comprehensive age-appropriate ophthalmic examination, including best-corrected visual acuity (BCVA) measurement with the Early Treatment Diabetic Retinopathy Study (ETDRS) charts, expressed as a logarithm of the minimum angle of resolution (logMAR), slit-lamp biomicroscopy, indirect ophthalmoscopy with 15 D noncontact lens (Volk), fluorescein angiography or color fundus photos (obtained by RetCam in younger patients requiring examination under anaesthesia and by Daytona wide-field retinography in compliant older subjects), optical coherence tomography (OCT), and full-field electroretinogram (ERG) recorded according to the ISCEV standards. Refractive errors ranged between +2 and −2 spherical equivalent. All examinations were performed at the referring clinic, and the results were collected at the end of the study.

Based on clinical data, each patient was classified according to the FEVR clinical staging system [16].

All patients received genetic counseling to explain the risks and benefits of genetic testing, and informed consent was obtained from their parents. The study followed the tenets of the Declaration of Helsinki.

Demographic details and information on personal and family medical history were recorded and used to determine the inheritance pattern and to draw pedigrees according to the recommendations of the National Society of Genetic Counselors [17] using the HaploPainter software [18].

Genetic testing was performed at MAGI's Laboratories (MAGI - Human Medical Genetics Institute, Rovereto and Bolzano, Italy). Extracted DNA (Blood DNA Kit E.Z.N.A.; Omega Bio-Tek Inc., Norcross, GA, USA) underwent polymerase chain reaction (PCR) to amplify all coding regions and the intron/exon junctions of the *FZD4* (OMIM ^∗^604579, NM_012193), *LRP5* (OMIM ^∗^603506, NM_002335), *TSPAN12* (OMIM ^∗^613138, NM_012338), and *NDP* (OMIM ^∗^300658, NM_000266) genes. Purified products were sequenced with a Beckman Coulter CEQ 8000 sequencer (Beckmann Coulter, Milano, Italy). All laboratory protocols are available on request.

The electropherograms of amplified fragments were analyzed using ChromasPro 1.5 (Technelysium Pty. Ltd., Australia) and Sequencher 5.0 (Gene Codes®; Ann Arbor, MI, USA) software and compared to GenBank reference sequences with the Basic Local Alignment Search Tool (BLAST; http://blast.ncbi.nlm.nih.gov). All detected genetic variations were sequenced bidirectionally at least twice for confirmation.

To identify variants previously reported as pathogenic, the Human Gene Mutation Database (HGMD; http://www.biobase-international.com/product/hgmd) was consulted. To evaluate Minor Allele Frequencies (MAF) in populations, all discovered genetic variants were also looked up in the public database of single-nucleotide variants (dbSNP; www.ncbi.nlm.nih.gov/SNP/) and the Exome Variant Server (EVS; http://evs.gs.washington.edu/EVS/) database. New nucleotide variations were assessed for pathogenicity using the PolyPhen 2 algorithm (Polymorphism Phenotyping v2; http://genetics.bwh.harvard.edu/pph2) considering the HumVar-trained model [19], the SIFT algorithm (Sorting Intolerant From Tolerant; http://sift.bii.a-star.edu.sg/) [20] and MutationTaster (http://www.mutationtaster.org) [21]. Defective splicing was evaluated using the Human Splicing Finder online software version 3 (http://www.umd.be/HSF3/HSF.html) [22]. When possible, wild-type amino acid properties were compared with the variations (http://www.russelllab.org/aas/aas.html) [23].

Variants were reported following the current nomenclature as described by the Human Genome Variation Society (http://www.hgvs.org/mutnomen/recs.html).

To classify sequence variants, we used the criteria by the American College of Medical Genetics and Genomics (ACMG) Standards and Guidelines [24].

## 3. Results

Genetic testing revealed that 6 out of 8 (75%) probands had a genetic variation in one of the genes analyzed. Consequently, a segregation study was performed by extending clinical and genetic analyses to a total of 10 relatives.

Pedigree analysis of patients with inherited forms of the disease led to the classification of four autosomal dominant cases of FEVR (*FZD4* and *TSPAN12* variations) and one X-linked FEVR proband (*NDP* variation) (see Table 1).

None of the patients showed variations in the *LRP5* gene.

No MAF are reported for the identified variants in all the questioned database.

In silico evaluation of genetic variations identified in FEVR families; genotype heterozygous (Het), hemizygous (Hemi); SIFT score system: tolerated (T), deleterious (D); Polyphen 2 score system: benign (B), possibly damaging (PoD), probably damaging (PrD); Mutation Taster score system: polymorphism (P), disease causing (DC); VUS: variation of unknown significance.

### 3.1. Family 1

The male proband (II:1) came to our attention only recently at the age of nine years.

**Figure 1:**
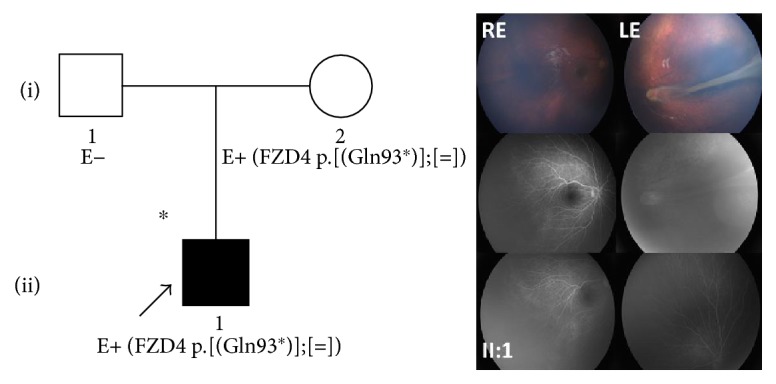
Pedigree and ocular features of family 1. Color fundus photographs by RetCam and fluorescein angiograms from the 9 y/o male proband (II:1) of family 1. The RE presented normal posterior pole; however, the fluorescein angiography showed a large area of avascular retina in the peripheral retina (FEVR stage 1). No signs of neovascularization or peripheral exudation were noticed. The LE presented a falciform retinal fold anchored to the temporal retinal sector without signs of peripheral exudation (FEVR stage 3A). ^∗^Documented clinical evaluation; E+ and E−, positive and negative to genetic test, respectively; RE, right eye; LE, left eye.

Previously performed fluorescein angiography with RetCam showed a large avascular area in the peripheral retina without signs of neovascularization or peripheral exudation in his right eye and a falciform retinal fold anchored to the temporal retinal sector without signs of peripheral exudation in the left eye (Figure 1). The patient was diagnosed with stage 1 and stage 3A FEVR in the right and left eyes, respectively. Clinical conditions are currently stable, and visual acuity is 0.0 LogMAR in the right eye and 1.0 LogMAR in the left eye. It was not possible to perform a complete clinical examination on the proband's mother (I:2) and father (I:1). The last fundus examination of the mother showed normal retinal features; however, we could not proceed with any instrumental examinations due to the patient's refusal to undergo further testing.

Genetic testing revealed that the proband and his mother were heterozygous for the new p.(Gln93^∗^) variation in the *FZD4* gene.

### 3.2. Family 2

This male proband (II:1) presented exotropia of the right eye at the age of 2 years. Ophthalmological examination under general anaesthesia showed inoperable falciform fold in the right eye and vascular alterations in the left eye. The patient was diagnosed with stage 3 and stage 2 FEVR in the right and left eyes, respectively. At the age of 3 years, scleral buckle, cryo treatment, and laser treatment for retinal detachment were performed in the right eye and laser treatment of the retinal periphery in the left eye. After a period of clinical stationary conditions, at the age of 5 years, the patient required further laser treatment in the left eye due to reactivation of the disease. Visual acuity is currently 0.1 and 0.2 LogMAR in the right and left eye, respectively.

The fundus examination of the father (I:1) showed slight abnormalities of the peripheral retina suggesting a condition of FEVR healthy carrier.

**Figure 2:**
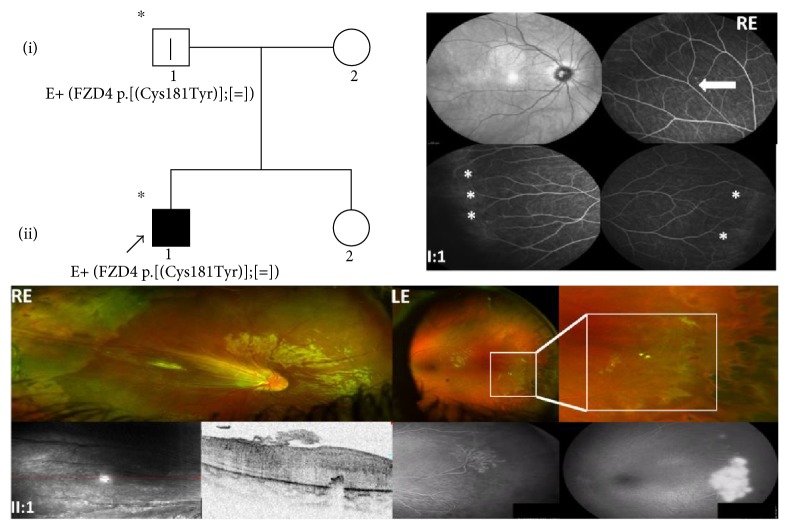
Pedigree and ocular features of family 2. Red-free images and fluorescein angiograms from the proband's father (I:1) showing similar findings in both eyes consisting of slight abnormalities of the peripheral retinal vasculature as indicated by the arrows. The posterior pole and the mid periphery were normal. Daytona wide-field color fundus photos and OCT scan of the proband (II:1) at the age of 2 years showing the presence of a falciform fold and peripheral avascular retina in the RE. The wide-field angiography of the LE (early and late phases) revealed a normal posterior pole and a severe leakage from undiscovered peripheral neovascular networks that were treated promptly to avoid exudation and visual impairment. ^∗^Documented clinical evaluation; E+ and E−, positive and negative to genetic test, respectively; ^|^asymptomatic/presymptomatic carrier (stage 1 FEVR); RE, right eye; LE, left eye.

Genetic testing revealed that the proband and his father were heterozygous for a variation of the gene *FZD4*, p.(Cys181Tyr); the mother was not assessed.  Figure 2 shows the autosomal dominant transmission of the variant p.(Cys181Tyr) in the *FZD4* gene and the clinical features of the family.

### 3.3. Family 3

The proband (II:2), a 19 y/o male, presented disease onset at the age of 13 years, with bilateral exudative retinopathy associated with slight visual acuity reduction. In detail, the patient showed exotropia of the right eye from early infancy and visual acuity of 0.1 LogMAR in both eyes. At the age of 13 years, he suffered a sharp decline in visual acuity (0.3 LogMAR in both eyes), associated with hard retinal exudates in the temporal periphery at the fundus examination. Based on the presence of posterior hyaloid contraction, macular edema, macular dragging, and accentuated peripheral exudation in both eyes, bilateral stage 4A FEVR was postulated. A few months after diagnosis, the patient underwent vitrectomy, cryo treatment, and injection of humanized anti-VEGF monoclonal antibody (Bevacizumab) in both eyes.

**Figure 3:**
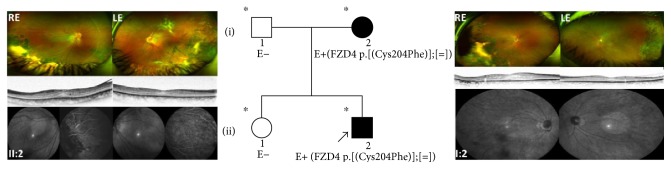
Pedigree and ocular features of family 3. Daytona wide-field color fundus photos and OCT scans of the proband (II:2) at the age of 19 years with bilateral exudative manifestation of FEVR stage 4A. Peripheral retinal signs of cryo treatment and Argon laser barrage, performed after evidence of fluorangiographic leakage, can be seen in the inferior temporal sectors in both eyes. Daytona wide-field color fundus photographs of the proband's mother (I:2), showing treatment with Argon laser in the retinal periphery of both eyes. Angiograms from the posterior pole and from the midperiphery do not present signs of disease's activity after laser treatment. ^∗^Documented clinical evaluation; E+ and E−, positive and negative to genetic test, respectively; RE, right eye; LE, left eye.

The patient was monitored every 6 months for the following three years by visual acuity measurements, OCT scans, and fluorescein angiographies. Since the peripheral exudations remained active, with angiographic evidence of leakage, the patient underwent multiple sessions of Argon laser, cryo treatment, and Bevacizumab injections (Figure 3). At the time of this report, visual acuity was 0.1 LogMAR in both eyes without signs of pathological activity for the last three years.

The proband's father (I:1) and sister (II:1) are healthy, whereas his mother (I:2), 52 years old, was diagnosed with stage 2B FEVR and related mild macular edema at the age of 46. Both eyes were treated with Argon laser, and her current visual acuity is 0.0 LogMAR in both eyes (Figure 3).

Autosomal dominant inheritance was suspected before genetic evaluation (Figure 3). Genetic evaluation showed that the proband and his mother were heterozygous for a new p.(Cys204Phe) variant in the FZD4 gene, while the father and the sister were negative to the test.

### 3.4. Family 4

**Figure 4:**
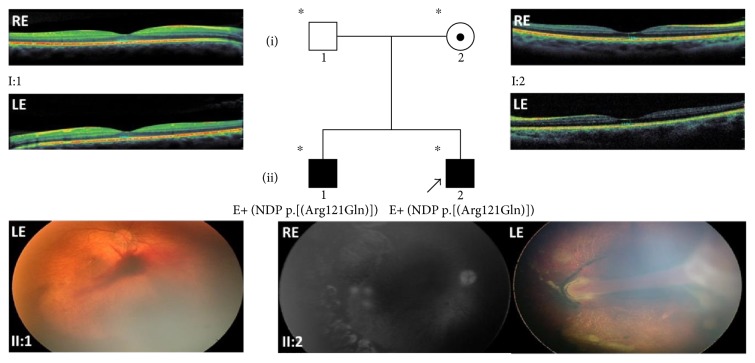
Pedigree and ocular features of family 4. Fluorescein angiograms by RetCam from the 7 y/o male proband (II:2) of family 4, showing FEVR stage 2B with slight optic nerve pallor, areas of chorioretinal atrophy due to previous laser treatment and a persisting small area of leakage in the peripheral retina of the RE. Color fundus photographs by RetCam of the LE with FEVR stage 3B clearly show the presence of a falciform fold arising from the optic nerve head, with partial traction on the surrounding retina. Stage 3A FEVR of the proband's older brother (II:1) is shown in the color fundus photograph from his LE. Macular OCT images of proband's father and mother are also shown in upper left and upper right quadrants of figure, respectively. The mother's exam shows a slight reduction of macular thickness. ^∗^Documented clinical evaluation; E+ and E−, positive and negative to genetic test, respectively; ^●^obligate carrier; RE, right eye; LE, left eye.

This 7 y/o male proband (II:2) came to our attention at the age of one year presenting congenital sensory nystagmus. Ophthalmological examination under sedation revealed the presence of a falciform fold arising from the optic nerve head, with partial traction on the surrounding retina in the left eye and slight optic nerve pallor with areas of chorioretinal atrophy due to previous laser treatment in the peripheral retina in the right eye. Consequently, stages 3B and 2B FEVR were diagnosed in the left and right eyes, respectively. A fluorescein angiography exam performed with RetCam showed a small area of hyperfluorescence next to the laser-treated areas, which required further laser treatment (Figure 4). At subsequent examinations, both eyes were stable. At the orthoptic examination, torticollis due to nystagmus with the face rotated to the right to favour fixation of the right eye in adduction was found. Visual acuity was 0.1 LogMAR in the left eye and 0.4 LogMAR in the right eye, respectively.

The proband's 10 y/o brother (II:1) was diagnosed with bilateral retinal dystrophy and falciform retinal fold at the age of three, suggesting a stage 3A FEVR (Figure 4). The left eye also showed a slight posterior opacity of the lens while the ectopic pupil was visible in the right eye. Visual acuity was lower than 0.1 LogMAR in both eyes.

The children's parents have visual acuities of 0.0 LogMAR in both eyes. The father's fundus was normal, whereas the mother showed slight mottling of the retinal pigmented epithelium, more evident at the periphery. Full-field electroretinography was within normal limits in the parents, though the mother's scotopic ERG responses were borderline and her macular OCT scans showed a reduction of foveal thickness suggesting a condition of asymptomatic carrier (Figure 4).

Genetic testing revealed the p.(Arg121Gln) variant in the *NDP* gene, already associated in literature with Norrie disease (MIM ^∗^310600) [25] (Figure 4).

### 3.5. Family 5

**Figure 5:**
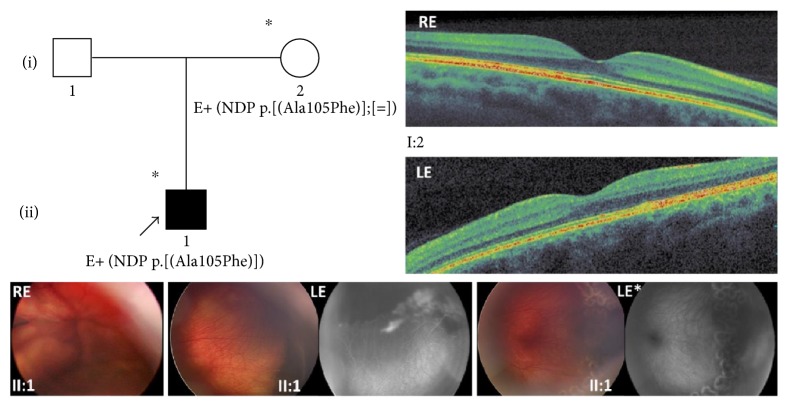
Pedigree and ocular features of family 5. Color fundus photographs by RetCam from the 3 y/o male proband (II:1) of family 5 showing a closed funnel total retinal detachment in the right eye and peripheral exudation with vascular abnormalities before and after laser treatment in the left eye. Fluorescein angiograms performed with RetCam before and after laser treatment showing peripheral ischemic areas with leakage mainly in temporal and inferior peripheral retina in the left eye. Top right, OCT macular scans from the proband's mother (I:2) showing normal retinal features. ^∗^Documented clinical evaluation; E+, positive to genetic test; RE, right eye; LE, left eye; LE^∗^, images taken at a subsequent examination after the laser treatment.

The 3 y/o male proband (II:1) came to our attention at the age of four months presenting leukocoria in the right eye. The fundus examination under sedation showed an inoperable macula-involving retinal detachment with subretinal exudate in the right eye and peripheral exudation with vascular abnormalities in the left eye. A fluorescein angiography exam performed with RetCam showed peripheral ischemic areas with leakage mainly in temporal and inferior peripheral retina which required laser treatment in the left eye. Consequently, stages 4B and 1B FEVR were diagnosed in the right and left eyes, respectively. At subsequent examinations, the right eye progressed into a closed funnel total retinal detachment whereas the left eye was stable. The child's father could not be examined while the mother showed a visual acuity of 0.0 LogMAR in both eyes. Her fundus was normal presenting only a slight mottling of the retinal pigmented epithelium in the peripheral retina. Full-field electroretinography and macular OCT scans were within normal limits in both eyes. (Figure 5). Genetic testing revealed that the proband and his mother (I:2) have a p.(Ala105Phe) variation in the *NDP* gene.

### 3.6. Family 6

**Figure 6:**
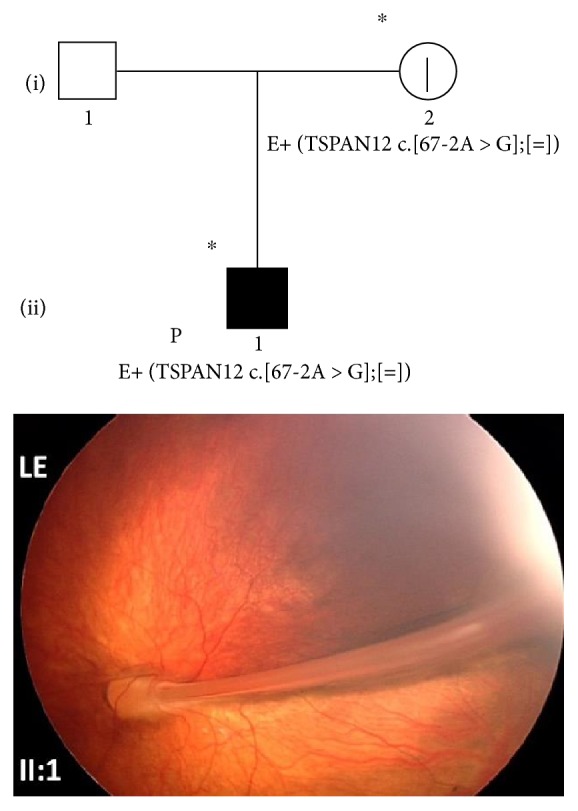
Pedigree and ocular features of family 6. RetCam color fundus photograph from the LE of the 7 y/o male proband (II:1) of family 6, showing FEVR stage 3A for the presence of a falciform retinal fold arising from the optic nerve head, involving the macula and anchored in the temporal periphery without signs of exudation. ^∗^Documented clinical evaluation; E+ and E−, positive and negative to genetic test, respectively. ^|^Asymptomatic/presymptomatic carrier (stage 1 FEVR); LE, left eye.

The 7 y/o male proband (II:1) was periodically monitored for visual loss due to FEVR since the age of one. The fundus examination under sedation showed a falciform retinal fold arising from the optic nerve head, involving the macula and anchored in the temporal periphery in his left eye (Figure 6). No exudation was evident near the fold or the temporal or nasal periphery. Based on aforementioned clinical data, stage 3A FEVR was postulated. No significant changes were found at the subsequent follow-up. The ophthalmological examination performed at the age of three showed residual visual acuity of 1.0 LogMAR in both eyes. The proband's father did not present any sign of ocular pathology while the mother was diagnosed with stage 1 FEVR in both eyes.

Genetic testing revealed the heterozygous IVS2 c.67-2A>G variant in the *TSPAN12* gene, and the same variant was found in the proband's mother (I:2), thus configuring an autosomal dominant transmission (Figure 6).

## 4. Discussion

In the present work, we reported the clinical findings and the genetic analysis from a cohort of 6 unrelated Italian families made up of 6 probands and 10 family members, thus presenting the results from the largest cohort of Italian FEVR families and data from four novel genetic variants linked to FEVR.

Retinal vascularization during eye development and maintenance of its normal architecture are processes finely orchestrated by the Norrin/*β*-catenin signaling pathway. This pathway is distinguished from the Wingless (Wnts) signaling pathways, which include a large family of ligands and are involved in many cell processes, and is specific for the development and maintenance of the retinal vasculature. The bond in NDP is in fact highly specific for the receptor complex FZD4-LRP5 [26] while TSPAN12 is required for FZD4/*β*-catenin signaling induced by Norrin. Moreover, TSPAN12 expression is restricted to the vasculature within the retina [27].

Aberrations and malfunctioning of this signaling system affect the development of the retinal vasculature, and this translates into a variety of eye diseases such as Norrie disease, FEVR, and retinopathy of prematurity [5, 6, 28]. All these disorders are clinically and genetically heterogeneous. Nucleotide alterations in the genes' coding for fundamental components of this signaling pathway, such as NDP growth factor (*NDP* gene), FZD4 receptor (*FZD4* gene), LRP5 coreceptor (*LRP5* gene), and tetraspanins (*TSPAN12* gene) are described as associated with different pathological phenotypes, ranging from mild to severe, even in the same family, or with clinically indistinguishable patterns caused by variations in different genes.

Our genetic testing investigated on the four genes most widely associated in literature with FEVR: *NDP*, *FDZ4*, *LRP5*, and *TSPAN12* [3].

In the present report, families 1, 2, and 3 had three different heterozygous variants in the *FZD4* gene. *FZD4* codes for FZD4, a member of the Frizzled family of seven-transmembrane Wnt-binding receptors. The binding of Wnt or Norrin ligands with FZD4 in conjunction with LRP5 coreceptors results in the activation of canonical *β*-catenin-dependent signaling. The N-terminal extracellular cysteine-rich domain (CRD), conserved among Frizzled family members, is necessary for binding to ligands [29].

The p.(Gln93^∗^) variation in the *FZD4* gene found in family 1 introduces a premature stop codon, and therefore, it is predicted to be pathogenic.

In families 2 and 3, the two variants are found in the extracellular N-terminal domain of the receptor, in amino acid positions 181 and 204, respectively. Both are presumably downstream of the CRD required for ligand binding, according to Smallwood et al. (i.e., the 114-amino acid region extending from the first to the tenth conserved CRD cysteine) [30], but their importance is relevant because variations in both codons, associated with a pathological phenotype, have already been described in literature and both can therefore be considered mutational hot spots.

The variant p.(Cys181Tyr) has already been described by Drenser et al. and associated with autosomal dominant FEVR [31]. The author concluded that this cysteine residue is not known to form an intracellular disulphide bond; it is nevertheless the 11th of 13 cysteine residues that are conserved in vertebrates and may be required for receptor dimerization [32], a function that may be relevant to the mechanism of Wnt binding and signaling [33]. In family 2, the healthy father, who carries this variant, showed a subclinical phenotype (stage 1 FEVR). It is well known that variations in this gene have complete (100%) penetrance but variable expression: members of the same family may have the same variant and show different severities of the disorder or may have retinal changes detectable only by diagnostic tools such as fluorescein angiography. Moreover, the disease phenotype in this family showed interesting aspects of FEVR pathology: (1) an asymmetrical impairment of the retina, (2) the importance of a follow-up in patients by fluorescein angiography, even in case of a normal (left eye) posterior pole, (3) the necessity of a different treatment approach for the lesions depending on the stages of the disease, and (4) the evidence that inactive lesions may later on constitute an ophthalmological emergency (retinal detachment in the right eye).

Similarly, variations in the 204 codon, as we found in family 3, have already been associated with autosomal dominant FEVR in two reports, one describing the amino acid change p.(Cys204Arg) [34] and the other, the p.(Cys204Tyr) change [35].

A functional study by Zhang et al. showed that FZD4 binding to NDP is disrupted by Cys204Arg, suggesting that the CRD may be beyond the previously predicted region, as described by Smallwood et al. [30], or that NDP binding to FZD4 requires the CRD plus additional residues, C-terminal to the CRD [29]. The autosomal dominant feature of *FZD4* variants could be due either to haploinsufficiency or to a dominant-negative effect, as previous findings suggest [36].

In family 4, the proband and the brother are affected by an X-linked form of FEVR due to a variant in the *NDP* gene. NDP is a protein ligand not belonging to the Wnt family which recognises and binds with high affinity and specificity to the CRD of FZD4 (and not to CRDs of the other 14 mammalian Frizzled and secreted Frizzled-related proteins) [30] and activates the canonical signaling pathway [26].

The p.(Arg121Gln) variant in the *NDP* gene has already been reported in literature, and the amino acid residue 121 seems to be a mutational hot spot. Indeed, the same amino acid change from arginine to glutamine has been associated with Norrie disease [25]; however, variants in the same codon, specifically p.(Arg121Gly) [37] and p.(Arg121Trp) [38], have been associated both with Norrie disease and with X-linked FEVR, that is, the p.(Arg121Leu) [39] and p.(Arg121Trp) variants [40].

Similarly, the p.(Ala105Phe) found in family 5 lies in a mutational hot spot since two other variants in the same codon, namely, the p.(Ala105Thr) and the p.(Ala105Glu), were described as associated with Norrie Disease [13] and FEVR [41], respectively.

Both variants found in families 4 and 5 are located in the C-terminal end of the Norrie protein, probably affecting the secondary structure and function of the protein.

Norrie disease is a complex disorder in which blindness in early childhood may be accompanied by sensorineural deafness and progressive mental retardation, inherited by X-linked recessive transmission. Heterozygous carriers rarely manifest clinical features of the disorder, though some cases have been described [42, 43]. The absence of a syndromic pattern with extraocular manifestations and preservation of some vision suggested a diagnosis of FEVR rather than Norrie disease, in both families.

Furthermore, the description of the same variant in subjects with different clinical patterns may indicate the involvement of other factors that might modify the phenotype.

In family 6, the proband and his mother harbour the same heterozygous variant IVS2 c.67-2A>G in the *TSPAN12* gene. TSPAN12 is a member of the tetraspanin superfamily characterized by four transmembrane domains. It is a key component of the NDP-FZD4-LRP5 signaling complex that cooperatively promotes multimerization of FZD4 and its associated proteins to elicit physiological levels of signaling [27]. Both dominant and recessive variants in *TSPAN12* have been described in FEVR [8, 11].

The novel variant reported here is predicted to interfere with the consensus sequence for the splice acceptor site of intron 2, as confirmed using HSF.

Poulter et al. described the c.67-1G>C variant in both homozygous and heterozygous states in two FEVR Indian cousins; the c.67-1G>G splice variant causes exon 3 deletion, resulting in a frameshift and a premature termination codon (p.Leu23GlyfsX66) [11]. Considering that the c.67-2A>G variant reported in our work involves the same acceptor splicing site, it is reasonable to expect the same pathogenetic effect.

The severe clinical expression of the disease observed in our patient is consistent with previous studies where variations in *TSPAN12* gene are reported to be associated with very severe disease phenotypes [11, 27].

## 5. Conclusions

To our knowledge, this is the largest cohort study of Italian FEVR patients. In the present retrospective study involving different clinical sites, the probands and their family members were clinically examined and identified by standard diagnostic tests, albeit performed by different operators; this, together with the limited observed population, impedes critical analysis of the phenotypic differences associated with the variations in different genes of the Norrin/Frizzled signaling pathway in our group of FEVR patients. Variations of the analyzed genes were found in 6 out of 8 (75%) patients, and four novel variants responsible for the phenotype were identified.

The variants in the *FZD4* gene found in families 1, 2, and 3 were associated with the mildest phenotype: in families 1 and 2, FEVR manifested with late onset and vision was preserved in the affected family members, whereas in family 3, patients showed earlier disease onset with relatively preserved vision in the proband and mild loss of visual acuity in the mother.

The X-linked FEVR variants involving the *NDP* gene in families 4 and 5 are considered severe because they determine an early onset of the disease with relevant retinal alterations. Minimal signs of retinal abnormalities were detected also in the mother of the two affected children who carried the same variant in family 4, while in family 5, the variant exerted no effects in the proband's mother.

The variant in the *TSPAN12* gene in family 6 caused a very severe phenotype that manifested from the first months of life with almost complete vision loss.

Our results are in agreement with the previously described literature confirming that familial exudative vitreoretinopathy presents a penetrance close to 100% [16] but is clinically and genetically heterogeneous [12], even in the same family [44]. Indeed, the two new heterozygous variants found in the *FZD4* gene and those in NDP and *TSPAN12* genes, respectively, were correlated with variable phenotypical presentation ranging from relatively mild to severe anatomical and functional impairment. Severity of phenotype was dependent on gene involvement and site of nucleotide variations.

Analysis of new genes recently found to be associated with FEVR will make it possible to improve the understanding of the pathogenesis of the disease.

## Figures and Tables

**Table 1:** Features of genetic variations found in FEVR families.

Family ID Gene	Genotype	Nucleotide change	Amino acid change	SIFT	Polyphen	Mutation taster	Classification	References
Fam. 1 *FZD4* NM_012193	Het	c.277C>T	p.(Gln93∗)	—	—	—	Pathogenic	Novel variant
Fam. 2 *FZD4* NM_012193	Het	c.542G>A	p.(Cys181Tyr)	T	PrD	DC	Pathogenic	[31]
Fam. 3 *FZD4* NM_012193	Het	c.611G>T	p.(Cys204Phe)	D	PrD	DC	Likely pathogenic	Novel variant
Fam. 4 *NDP* NM_000266	Hemi	c.362G>A	p.(Arg121Gln)	D	PrD	DC	Pathogenic	[25]
Fam. 5 *NDP* NM_000266	Hemi	c.313G>C	p.(Ala105Phe)	D	PrD	DC	Likely pathogenic	Novel variant
Fam. 6 *TSPAN12* NM_012338	Het	c.67-2A>G	Defective splicing	—	—	—	Likely pathogenic	Novel variant
