# Corrigendum to “Genotype-Phenotype Characterization of Novel Variants in Six Italian Patients with Familial Exudative Vitreoretinopathy”

**DOI:** 10.1155/2017/7969364

**Published:** 2017-11-30

**Authors:** Giancarlo Iarossi, Matteo Bertelli, Paolo Enrico Maltese, Elena Gusson, Giorgio Marchini, Alice Bruson, Sabrina Benedetti, Sabrina Volpetti, Gino Catena, Luca Buzzonetti, Lucia Ziccardi

**Affiliations:** ^1^Department of Ophthalmology, Bambino Gesù IRCCS Children's Hospital, Rome, Italy; ^2^MAGI-Human Medical Genetics Institute, Bolzano, Italy; ^3^MAGI-Human Medical Genetics Institute, Rovereto, Italy; ^4^Eye Clinic, Department of Neurosciences, Biomedicine and Movement, University and AOUI (Azienda Ospedaliera Universitaria Integrata) of Verona, Verona, Italy; ^5^Dipartimento Anestesia e Rianimazione Materno Infantile, Ospedale San Filippo Neri, Rome, Italy; ^6^“G.B. Bietti” Foundation, IRCCS, Rome, Italy

In the article titled “Genotype-Phenotype Characterization of Novel Variants in Six Italian Patients with Familial Exudative Vitreoretinopathy” [1], there was an error in the variation in the *NDP* gene c.313G>C. Accordingly, “p.(Ala105Phe)” should be corrected to “p.(Ala105Pro)” in the Results and Discussion. Also, Table 1 and Figure 1 should be corrected as follows:

## Figures and Tables

**Figure 1:**
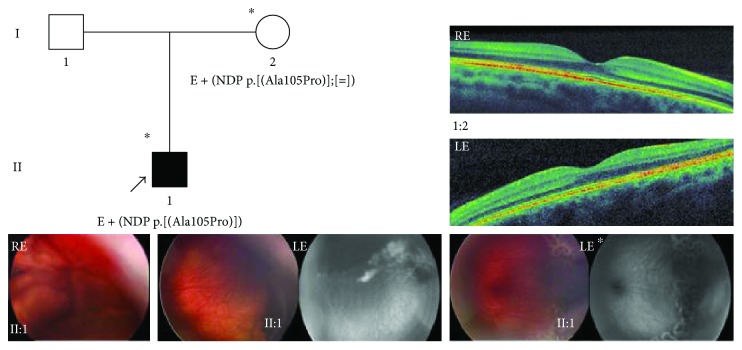
Pedigree and ocular features of family 5. Color fundus photographs by RetCam from the 3 y/o male proband (II:1) of family 5 showing a closed funnel total retinal detachment in the right eye and peripheral exudation with vascular abnormalities before and after laser treatment in the left eye. Fluorescein angiograms performed with RetCam before and after laser treatment showing peripheral ischemic areas with leakage mainly in temporal and inferior peripheral retina in the left eye. Top right, OCT macular scans from the proband's mother (I:2) showing normal retinal features. ^∗^Documented clinical evaluation; E+, positive to genetic test; RE, right eye; LE, left eye; LE^∗^, images taken at a subsequent examination after the laser treatment.

**Table 1:** Features of genetic variations found in FEVR families.

Family ID Gene	Genotype	Nucleotide change	Amino acid change	SIFT	Polyphen	Mutation taster	Classification	References
Fam. 1 *FZD4* NM_012193	Het	c.277C>T	p.(Gln93^∗^)	—	—	—	Pathogenic	Novel variant
Fam. 2 *FZD4* NM_012193	Het	c.542G>A	p.(Cys181Tyr)	T	PrD	DC	Pathogenic	[31]
Fam. 3 *FZD4* NM_012193	Het	c.611G>T	p.(Cys204Phe)	D	PrD	DC	Likely pathogenic	Novel variant
Fam. 4 *NDP* NM_000266	Hemi	c.362G>A	p.(Arg121Gln)	D	PrD	DC	Pathogenic	[25]
Fam. 5 *NDP* NM_000266	Hemi	c.313G>C	p.(Ala105Pro)	D	PrD	DC	Likely pathogenic	Novel variant
Fam. 6 *TSPAN12* NM_012338	Het	c.67-2A>G	Defective splicing	—	—	—	Likely pathogenic	Novel variant
